# Reporting Quality of Journal Abstracts for Surgical Randomized Controlled Trials Before and After the Implementation of the CONSORT Extension for Abstracts

**DOI:** 10.1007/s00268-019-05064-1

**Published:** 2019-06-20

**Authors:** Benjamin Speich, Kimberly A. Mc Cord, Arnav Agarwal, Viktoria Gloy, Dmitry Gryaznov, Giusi Moffa, Sally Hopewell, Matthias Briel

**Affiliations:** 1 Centre for Statistics in Medicine, Nuffield Department of Orthopaedics, Rheumatology and Musculoskeletal Sciences, University of Oxford, Oxford, UK; 2 Basel Institute for Clinical Epidemiology and Biostatistics, Department of Clinical Research, University Hospital Basel and University of Basel, Basel, Switzerland; 3 Department of Health Research Methods, Evidence, and Impact, McMaster University, Hamilton, ON Canada; 4 School of Medicine, University of Toronto, Toronto, ON Canada; 5 Department of Health Research Methods, Evidence, and Impact, McMaster University, Hamilton, Canada

## Abstract

**Background:**

Adequate reporting is crucial in full-text publications but even more so in abstracts because they are the most frequently read part of a publication. In 2008, an extension for abstracts of the Consolidated Standards of Reporting Trials (CONSORT-A) statement was published, defining which items should be reported in abstracts of randomized controlled trials (RCTs). Therefore, we compared the adherence of RCT abstracts to CONSORT-A before and after the publication of CONSORT-A.

**Methods:**

RCTs published in the five surgical journals with the highest impact factor were identified through PubMed for 2005–2007 and 2014–2016. Adherence to 15 CONSORT-A items and two additional items for abstracts of non-pharmacological trials was assessed in duplicate. We compared the overall adherence to CONSORT-A between the two time periods using an unpaired *t* test and explored adherence to specific items.

**Results:**

A total of 192 and 164 surgical RCT abstracts were assessed (2005–2007 and 2014–2016, respectively). In the pre-CONSORT-A phase, the mean score of adequately reported items was 6.14 (95% confidence interval [CI] 5.90–6.38) and 8.11 in the post-CONSORT-A phase (95% CI 7.83–8.39; mean difference 1.97, 95% CI 1.60–2.34; *p* < 0.0001). The comparison of individual items indicated a significant improvement in 9 of the 15 items. The three least reported items in the post-CONSORT-A phase were randomization (2.4%), blinding (13.4%), and funding (0.0%). Specific items for non-pharmacological trials were rarely reported (approximately 10%).

**Conclusion:**

The reporting in abstracts of surgical RCTs has improved after the implementation of CONSORT-A. More importantly, there is still ample room for improvement.

**Electronic supplementary material:**

The online version of this article (10.1007/s00268-019-05064-1) contains supplementary material, which is available to authorized users.

## Introduction

There is great agreement that randomized controlled trials (RCTs) produce the most reliable evidence about the benefits and risks of newly developed or already existing clinical interventions, ultimately leading to better care for patients [1–3]. To allow for informed judgments about the external validity and methodological quality of RCTs, adequate reporting is of uttermost importance [4]. For published RCTs, a number of research studies have identified serious limitations in reporting [3, 5–9].

Over the last decade, there have been considerable efforts to improve the quality of reporting of individual research studies. Commonly this challenge has been tackled with the development of reporting guidelines which provide structured advice on the minimum information needed in a research article to allow readers an adequate assessment of the study methodology, relevance and validity of the research findings. The last update of the Consolidated Standards of Reporting Trials (CONSORT) Statement, the most important reporting guideline intended to improve the transparency and quality of RCT reporting, was published simultaneously in 10 leading medical journals in 2010 [10].

Despite a certain improvement with implementation of the CONSORT Statement, there still remain major reporting deficiencies in published RCTs [11]. Compared to drug trials, surgical RCTs face several specific challenges (e.g. learning curve, high proportion of crossover due to preference and lack of blinding) [12] and the quality of reporting in surgical trials is particularly low [13]. Therefore, Boutron and colleagues developed an extension to the CONSORT Statement specifically for reporting trials of surgical and non-pharmacological treatments (CONSORT-NPT) [14, 15].

Poor reporting is not only an issue in full-text publications but also in journal abstracts as well as in conference abstracts [16]. This can have great implications because the abstract is often the only part of an article which is read by clinicians due to time constraints or because the full-text publication is not freely available [17]. Therefore, poor reporting can lead to wrong decisions in clinical practice. Henceforth, the CONSORT group developed and published in 2008 a CONSORT extension, specifically for reporting the abstracts of RCTs (CONSORT-A) [18]. Within the present study, we aim to evaluate the quality of reporting in journal abstracts of RCTs in the top ranked surgical journals before CONSORT-A was published and after publication.

## Methods

### Search strategy and inclusion criteria

We searched PubMed for RCTs published in the years 2005–2007 (before the CONSORT-A extension was published) and 2014–2016 (after the CONSORT-A extension was published) in the five surgical journals with the highest impact factor in 2017 (according to the Thomson Reuters InCites Journal Citation Reports [19]; the detailed search strategy is provided in the supplementary Appendix). We did not consider journals which were listed among the top journals but were founded after 2005. We included primary reports of RCTs (i.e. those reporting on the primary outcome) which evaluated surgical procedures as well as other interventions if they were clearly associated with a surgical procedure (i.e. directly before or after surgery: e.g. physical exercise before surgery, diet intervention after surgery). We excluded articles which were not about an RCT, performed additional, secondary analyses of an RCT or a study within a trial (SWAT), explicitly labeled pilot and feasibility studies, and in case the RCT had nothing to do with surgery or was not conducted directly before, during or after surgery (e.g. evaluation of new treatments 1 year after organ transplantation). Articles on RCTs were also excluded if the time point of the outcomes did not clearly include the primary outcome (e.g. additional long-term results 10 years after surgery). Two reviewers independently screened titles and abstracts for eligibility (BS, KAM, AA, VG, DG and MB). The full text was only considered when it remained unclear if the article should be included based on the abstract. Disagreements were resolved by discussion.

### Data extraction

Two reviewers independently extracted data from each included abstract. Each data extractor received a manual explaining the inclusion criteria and the specific items to extract. The extraction was first pilot tested to ensure that data extractors applied the same judgement on the different CONSORT-A items. A total of 15 items from the CONSORT-A [18] were assessed in duplicate if they were reported adequately (yes/no). We used the CONSORT explanation and elaborations, published by Hopewell and colleagues [18], to judge if an item was adequately reported [14]. The two conference abstract specific items “authors” and “recruitment” were not considered. To assess whether the blinding status was adequately reported, we copied the statement about blinding. In a first scenario, blinding was only assumed to be adequate if the status of involved persons (i.e. care provider, patients, outcome assessors) was clearly mentioned. In a second, less strict scenario, we also accepted general terms like “double-blind” or “single-blind”. Furthermore, whenever at least one of the treatment arms included a non-pharmacological intervention, the two items for abstracts of non-pharmacological abstracts were assessed according to an extension published by Boutron and colleagues [14].

### Sample size calculation

The objective of this study was to compare the number of reported items in surgical trials before and after the publication of the CONSORT extension for abstracts guidelines in 5 relevant journals. A previous study analyzing major clinical journals reported a mean difference of around 3 on the number of reported items (mean 2007: 9.06; standard deviation [SD] 2.15; mean 2012: 12.11; SD 2.22) with a standard deviation above 2 [20]. This study conducted by Mbuagbaw and colleagues assessed all 17 CONSORT-A items. When only looking at the same 15 items which we plan to assess, the average score by Mbuagbaw et al., would be 8.0 (2007) and 11.0 (2012). From a practical perspective, it would be of interest to detect mean differences as low as 1.5 items. Making the conservative assumption of a standard deviation of 2.5, we needed 60 articles per study period to have 90% power to detect a mean difference of 1.5 at significance level 5% using a *t* test. Because we had no a priori information about the distribution of articles across different journals and because of the rather descriptive character of this study, we did not directly consider the clustering before conducting the study and decided to include all relevant articles. This provided us with an inflated sample with respect to that needed under the assumption of independence; however, we had to account for the fact that with clustered data we need to inflate the sample size by the variance inflation factor (post hoc design analyses with different scenarios are presented within the supplementary Appendix) [21].

### Outcome measures and statistical analysis

The primary outcome was the mean difference, with 95% confidence intervals (CIs), in the frequency of adequate reporting of the 15 CONSORT items, meaning the difference of overall scores between the two time periods (2005–2007 vs. 2014–2016). Student’s unpaired *t* test was used to compare means. To assess whether an increase in frequency of adequate reporting may be simply explained by a time trend, we visualized box plots by year of publication. In addition we modeled the overall probability of reporting an item by means of a mixed effects logistic regression. For each article, we considered binary outcomes for all items (reported/not reported) and fitted a model with both a random effect for journal and a random effect for item, while adjusting for the sample size of each study. Three RCTs with an unclear sample size were excluded in that model. Secondary outcomes were the frequency of adequate reporting of separate CONSORT-A items, which we compared by means of Chi-squared tests and calculating odds ratios. Reporting of the two items for abstracts of non-pharmacological abstracts was presented descriptively.

## Results

**Fig. 1:**
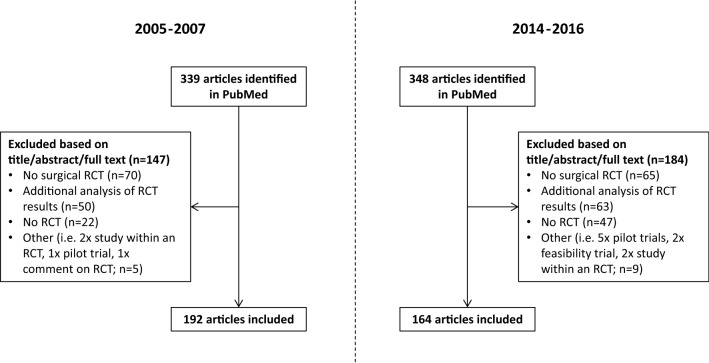
Flow chart

We identified 339 potentially eligible articles in the pre-CONSORT-A phase and 348 in the post-CONSORT-A phase (Fig. 1). Of those, a total of 192 (2005–2007) and 164 (2014–2016) were eligible to be included. The vast majority (274 of 356; 77.0%) of the included articles came from two journals (Annals of Surgery and British Journal of Surgery). The median sample size in RCTs in the included abstracts was 106 (interquartile range [IQR] 68–200) for the pre-CONSORT-A phase and 130 (IQR 80–240) for the post-CONSORT-A phase (Table 1). In most cases, the abstract did not contain information to determine whether the RCT was a single-center or multicenter study (overall unclear in 70.8%; Table 1).

**Table 1:** Baseline characteristics

	RCTs published from 2005 to 2007	RCTs published from 2014 to 2016	Overall
Included studies	192	164	356
Annals of surgery	63 (32.8%)	80 (48.8%)	143 (40.2%)
British journal of surgery	79 (41.4%)	52 (31.7%)	131 (36.8%)
American Journal Of Transplantation	37 (19.3%)	23 (14.0%)	60 (16.9%)
The journal of heart and lung transplantation	12 (6.3%)	6 (3.7%)	18 (5.1%)
Journal of neurology, neurosurgery, and psychiatry	1 (0.5%)	3 (1.8%)	4 (1.1%)
Median number of patients (IQR)^a^	106 (68–200)	130 (80–240)	120 (72–219)
Single-center or multicenter			
Single center	8 (4.2%)	20 (12.2%)	28 (7.9%)
Multicenter	28 (14.6%)	48 (29.3%)	76 (21.3%)
Unknown	156 (81.3%)	96 (58.5%)	252 (70.8%)

*RCT* randomized controlled trial, *IQR* interquartile range

^a^A total of 3 RCTs contained no information about sample size (2005–2007, *n* = 0; 2014–2016, *n* = 3)

**Fig. 2:**
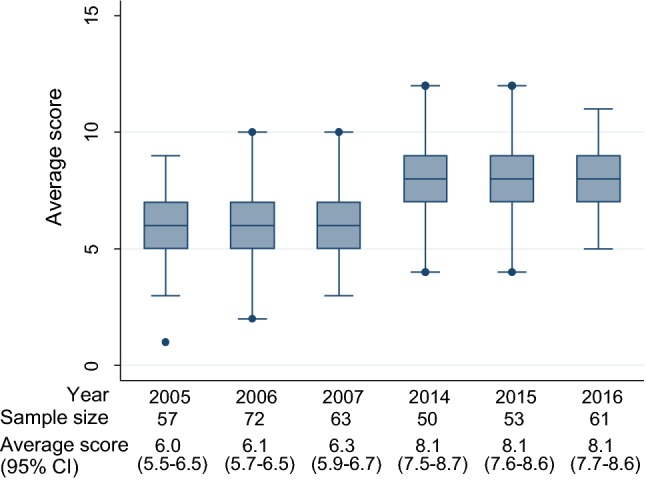
Mean score of adequate reporting by year in journal abstracts of surgical randomized controlled trials according to the CONSORT extension for abstracts. CI, confidence interval

The mean overall score of adequately reported CONSORT-A items was 6.14 (95% CI 5.90–6.38) for journal abstracts published between 2005 and 2007 and 8.11 (95% CI 7.83–8.39) from 2014 until 2016 (Table 2). The mean difference in overall score was 1.97 (95% CI 1.60–2.33; *p* < 0.0001). Inspection of boxplots for individual years did not show a general increasing trend over time (Fig. 2). From the random effects model (adjusted for journal, sample size and item), we got an odds ratio of 2.59 (95% CI 2.22, 3.03).

**Table 2:** Frequency of reporting in journal abstracts of surgical randomized controlled trials according to the CONSORT extension for abstracts

	RCTs published from 2005 to 2007	RCTs published from 2014 to 2016	Odds ratio (95% CI)	Mean difference (95% CI)	*p* value
Included studies	192	164			
Reporting in journal abstract					
Title: Identification of the study as randomized	160 (83.3%)	154 (93.9%)	3.08 (1.41–7.25)	–	0.0021
Trial design: description of the trial design	130 (67.7%)	133 (81.1%)	2.04 (1.21–3.48)	–	0.0042
Participants: eligibility criteria for the participants and the setting where the data were collected	21 (10.9%)	34 (20.7%)	2.12 (1.14–4.05)	–	0.0108
Interventions intended for each group	163 (84.9%)	151 (92.1%)	2.07 (1.00–4.49)	–	0.0364
Specific objective or hypothesis	148 (77.1%)	153 (93.3%)	4.14 (2.00–9.20)	–	<0.0001
Clearly defined primary outcome	54 (28.1%)	101 (61.6%)	4.10 (2.56–6.56)	–	<0.0001
Randomization: how participants were allocated to interventions	1 (0.5%)	4 (2.4%)	4.82 (0.47–238.3)	–	0.1229
Blinding: whether or not participants, caregivers, and those assessing the outcomes were blinded to group assignment	19 (9.9%)	22 (13.4%)	1.41 (0.70–2.87)	–	0.2999
^a^Blinding: term like “single-blind”, “double-blind”, “blinded” without further definition also accepted	44 (22.9%)	55 (33.5%)	1.70 (1.04–2.79)	–	0.0258
Number of participants randomized to each group	100 (52.1%)	97 (59.2%)	1.33 (0.86–2.07)	–	0.1815
Number of participants analyzed in each group	38 (19.8%)	51 (31.1%)	1.83 (1.09–3.06)	–	0.0141
Outcome: for the primary outcome, a result for each group and the estimated effect size and its precision	43 (22.4%)	85 (51.8%)	3.73 (2.30–6.05)	–	<0.0001
Harms: important adverse events or side effects	110 (57.3%)	107 (65.2%)	1.40 (0.89–2.20)	–	0.1253
Conclusion: general interpretation of the results	188 (97.9%)	162 (98.8%)	1.72 (0.24–19.25)	–	0.5280
Trial registration: registration number and name of trial register	4 (2.1%)	76 (46.3%)	40.59 (14.39–156.08)	–	<0.0001
Funding: source of funding	0 (0%)	0 (0%)	Not estimable	–	–
Average score (95% CI)	**6.14 (5.90**–**6.38%)**	**8.11 (7.83**–**8.39%)**	–	1.97 (1.60–2.33)	<0.0001
^a^Sensitivity analysis: average score (95% CI)	6.27 (6.02–6.52%)	8.31 (8.03–8.59%)	–	2.04 (1.67–2.41)	<0.0001

Average score of adequatley reported CONSORT-A items (main finding) are given in bold

*CI* confidence interval

^a^In the sensitivity analyses general terms like “single-blind”, “double-blind”, “blinded” were considered as adequate reporting

The overall increase in adherence to CONSORT-A was also visible at the level of individual items. Our exploratory analysis indicated a significant increase in 9 out of the 15 assessed items (Table 2). The items without significant improvement were randomization (adherence: 2005–2007, 0.5%; 2014–2016, 2.4%), number of participants randomized to each group (2005–2007, 52.1%; 2014–2016, 59.2%), harms (2005–2007, 57.3%; 2014–2016, 65.2%), funding (2005–2007, 0.0%; 2014–2016, 0.0%), blinding (2005–2007, 9.9%; 2014–2016, 13.4%), as well as conclusion (2005–2007, 97.9%; 2014–2016, 98.8%) which had already a very high adherence in the years 2005–2007. When ambiguous terms such as “double-blind” or “single-blind” were accepted, there was a significant increase in reporting of blinding status (2005–2007, 22.9%; 2014–2016, 33.5%; Table 2). Even though the adherence to CONSORT-A was substantially higher after the publication of the CONSORT extension for abstracts [18], there still remains 7 items which had an adherence below 50% in the 164 RCTs published in the post-CONSORT-A phase. These were about participants (20.7%), randomization (2.4%), blinding (13.4%), number of participants analyzed (31.1%), trial registration (46.3%), and funding (0.0%; Table 2).

The two adapted CONSORT-A items for non-pharmacological treatment were applicable for 116 RCTs in the time period from 2005 until 2007 and for 106 RCTs from 2014 until 2016. The reporting of those two items was low (approximately 10%) in both time periods (Table 3).

**Table 3:** Reporting of the two extension items for non-pharmacological treatments in abstracts of surgical randomized controlled trials

	RCTs published from 2005 to 2007	RCTs published from 2014 to 2016
Total included randomized controlled trials	192	164
Randomized controlled trials eligible for the non-pharmacological extension (at least one treatment arm with a non-pharmacological intervention)	116	106
Reporting of additional item 1: “When applicable, report eligibility criteria for centers where the intervention is performed and for care providers” [13]	11 (9.5%)	11 (10.5%)
Reporting of additional item 2: “Report any important changes to the intervention delivered from what was planned“ [13]	14 (12.1%)	10 (9.5%)

## Discussion

To our knowledge, this is the first systematic assessment of the reporting quality in surgical RCT abstracts according to CONSORT-A. We found a significant improvement in reporting of CONSORT-A items within surgical abstracts when comparing the time periods before and after the publication of the CONSORT for abstracts extension. However, the adherence to CONSORT-A still remains unsatisfying in 2014–2016 with a mean adherence of 8.11 items (15 assessed items in total). Looking at the individual items, there were four items which had a high or at least relatively high adherence already in the pre-CONORT-A phase (i.e. ≥ 70%). These were the following: title, intervention, specific objective or hypothesis, and conclusion. After the publication of the CONSORT-A extension, only one additional item reached at least 70% adherence (i.e. trial design). The increase in the overall score can probably be better explained when looking at the specific items which were rarely reported (below 30%). In surgical RCTs published between 2005 and 2007, a total of 9 CONSORT-A items were reported in less than 30% of the assessed abstracts (i.e. participants, clear defined primary outcome, randomization, blinding, number of participants analyzed, outcome, trial registration, funding). In the post-CONSORT-A phase, this number of highly underreported items was reduced to four (i.e. participants [20.7%], randomization [2.4%], blinding [13.4%], funding [0.0%]). Our two assessed scenarios of the blinding status showed that, if at all reported, mostly ambiguous terms such as “double-blind” are used instead of clearly mentioning the blinding status of involved individuals [22].

Adequate reporting seems to be neglected also in other medical fields. A similar study conducted by Mbuagbaw and colleagues [20] assessed the adherence to CONSORT-A in 2007 and 2012 in the five general medical journals with the highest impact factors. They also found a significant increase in the overall reporting of CONSORT-A items. The overall adherence in 2012 was 12.11 (out of 17 assessed items; 71.2%). Comparing this to our results (mean adherence of 8.11 in 2014–2016; a total of 15 assessed items; 54.1%), this might indicate that there is even larger room for improvement in adhering to CONSORT-A in surgical abstracts. This finding would be in agreement with several reporting assessments of the CONSORT statement (not CONSORT-A), which found that a factor associated with poor adherence was non-pharmacological trials [23]. Another study by Sriganesh et al. evaluated the adherence to CONSORT-A, before and after the publication of CONSORT-A, in the five pain journals with the highest impact factor. They found an improvement from a mean number of reported items of 6.12 in the years 2005–2007 (*n* = 125) to a mean number of reported items of 7.06 in the years 2013–2015 (*n* = 125) [24]. The reporting of several separate items was in a similar range as found in our study. For example, funding was also not reported within a single abstract in pain journals and the randomization was only reported in 2.4% (surgery also 2.4%) of abstracts in the post-CONSORT-A phase. Another two assessments of CONSORT-A in specialized medical fields (anesthesia and oral implantology journals) found similar results as well [25, 26].

Our study has the following strengths: We used a systematic approach in the frame of a before–after study design to assess whether the adherence to CONSORT-A improved. Inclusion criteria and data extraction were clearly defined and pilot tested. The screening of abstracts for inclusion as well as the data extraction was conducted in duplicate and all discrepancies were resolved by discussion. Each extractor assessed the same amount of abstracts in the pre-CONSORT-A phase and the post-CONSORT-A phase to make sure that the results are not influenced by individual judgments from data extractors. Our model-based analysis indicated a strong improvement even after accounting for clustering at the journal and item level, and adjusting for the study sample size. Moreover, there was no evidence of a gradual improvement over time suggesting the introduction of CONSORT-A as a plausible reason for the observed effect. We also assessed two specific items for non-pharmacological treatments highlighting that they are in general rarely reported.

There are a number of limitations worth mentioning. First, even though explanation and elaboration papers try to explain and standardize correct reporting, the assessment of adequate reporting always includes judgment. We tried to standardize our assessment by pilot testing, devising a manual, and extracting items in duplicate. For example, we did not request the term “parallel” or “superiority” when this was in general clear from the description. Second, some items consist of several aspects which were only judged in general and not for each specific aspect. The item “participants” was for example mostly not adequately reported because this item also requests a description of the study setting [18]. Third, the data extractors were aware if they extracted data from an abstract of the pre- or post-CONSORT-A phase. Therefore, they were not blinded which could have influenced the result. Fourth, the vast majority of included RCTs came from two surgical journals, Annals of Surgery and British Journal of Surgery. According to their author instructions, both of these journals allow only 250 words within abstracts which is rather short and might have influenced the adherence to CONSORT-A. Fifth, even though we also conducted an adjusted analysis, we could not account for other characteristics which may also explain a general improvement (e.g. funding source, number of centers, positive or negative results, journal, and endorsement of CONSORT-A guidelines). However, we are convinced that this limitation does not influence our main conclusions. This evaluation indicated clearly that reporting in surgical abstracts improved (underlining reason for that improvement can not be entirely assessed) and, more importantly, that adequate reporting is still relatively low. It is important that researchers are aware of the required information when presenting their results, so that readers can adequately and transparently judge the quality of the study. Journals with their editors play a crucial role in improving the abstract reporting. For example stating in the authors instructions that manuscripts should adhere to CONSORT-A. From the assessed journals, only the British Journal of Surgery clearly mentions in the authors instructions that CONSORT-A should be considered. The American Journal of Transplantation provides a link the EQUATOR (Enhancing the QUAlity and Transparency Of health Research) network encouraging to apply the appropriate guidelines [27]. Other options to improve adequate reporting in abstracts could consist of actively requesting adherence to missing items during the peer-review process and of less strict word limits for RCTs.

In conclusion, the adherence to CONSORT-A improved significantly when comparing the phase before CONSORT-A was published (i.e. years 2005–2007) with the post-CONSORT-A phase (i.e. years 2014–2016). However, the overall adherence remained unsatisfying and certain items were hardly ever adequately reported (i.e. randomization, blinding, funding).

## Electronic supplementary material

Below is the link to the electronic supplementary material.

Supplementary material 1 (DOCX 19 kb)
