# Supplementary material for: Reporting Quality of Journal Abstracts for Surgical Randomized Controlled Trials Before and After the Implementation of the CONSORT Extension for Abstracts
Source: World J Surg. 2019 Jun 20;43(10):2371–8. doi: 10.1007/s00268-019-05064-1 (PMC6722149; doi:10.1007/s00268-019-05064-1)
Supplement: Supplementary file 1 — Supplementary material 1 (DOCX 19 kb) [file 268_2019_5064_MOESM1_ESM.docx]

**Appendix**

**Reporting quality of journal abstracts for surgical randomized controlled trials before and after the implementation of the CONSORT extension for abstracts**

Benjamin Speich, Kimberly A. Mc Cord, Arnav Agarwal, Viktoria Gloy, Dmitry Gryaznov, Giusi Moffa, Sally Hopewell, Matthias Briel,

**Search strategy**

Search conducted on 17 July 2017.

Search on PubMed for time period 2005-2007:

((("Annals of surgery"[Journal] OR "Journal of neurology, neurosurgery, and psychiatry"[Journal]) OR "The British journal of surgery"[Journal]) OR "American journal of transplantation : official journal of the American Society of Transplantation and the American Society of Transplant Surgeons"[Journal]) OR "The Journal of heart and lung transplantation : the official publication of the International Society for Heart Transplantation"[Journal] AND (Randomized Controlled Trial[ptyp] AND ("2005/01/01"[PDAT] : "2007/12/31"[PDAT]))

Search on PubMed for time period 2014-2016:

((("Annals of surgery"[Journal] OR "Journal of neurology, neurosurgery, and psychiatry"[Journal]) OR "The British journal of surgery"[Journal]) OR "American journal of transplantation : official journal of the American Society of Transplantation and the American Society of Transplant Surgeons"[Journal]) OR "The Journal of heart and lung transplantation : the official publication of the International Society for Heart Transplantation"[Journal] AND (Randomized Controlled Trial[ptyp] AND ("2014/01/01"[PDAT] : "2016/12/31"[PDAT]))

**Post hoc sample size calculation with different scenarios**

In order to evaluate the sensitivity of the results to the variability of the design properties, we performed a post-hoc design analysis ^1^ where we considered different scenarios for the cluster size and its variance with respect to the number of articles actually extracted. We did not use any information about the estimated eﬀect and its standard deviation in the design analysis. For our sample of articles we found an average cluster size of 35.6 and a standard deviation of 33.0, so that the coefficient of variation is estimated as 0.93. Following Germini and colleagues ^2^ we assumed an intra-class correlation coefficient of 0.03. The power of a study with these design characteristics, and accounting for the clustering as suggested in Hemming et al.,^3^ is 90.6%. However, if we allowed for example for a standard deviation of 43 for the cluster size the power would reduce to about 84%, and if we considered a more extreme case where the standard deviation of the effect is 3 rather than 2.5, the power decreased to about 70%. Similarly if we had smaller cluster sizes on average the power would drop. If we consider the more extreme situation where we wish to detect a diﬀerence of 1.5 points, and we assume a standard deviation of 3, then the power available is about 78%. By bootstrapping we ﬁnd that the standard deviation of the standard deviation of the cluster size is approximately 5. We can use this information to consider alternative scenarios, with the standard deviation ranging in the interval (σ−10,σ +10). In the extreme case where the standard deviation of the cluster size is as high as σ +10 = 42.9818132, and the standard deviation of the eﬀect is assumed to be 3, the power reduces to about 70%.

1. Gelman A, Carlin J. Beyond Power Calculations: Assessing Type S (Sign) and Type M (Magnitude) Errors. *Perspect Psychol Sci* 2014; 9(6):641-51.

2. Germini F, Marcucci M, Fedele M, et al. Quality of reporting in abstracts of RCTs published in emergency medicine journals: a protocol for a systematic survey of the literature. *BMJ Open* 2017; 7(4):e014981.

3. Hemming K, Girling AJ, Sitch AJ, et al. Sample size calculations for cluster randomised controlled trials with a fixed number of clusters. *BMC Med Res Methodol* 2011; 11:102.
